# A Case Report of Delayed, Severe, Paroxysmal Muscle Cramping After Chilean Rose Tarantula *(Grammostola rosea*) Envenomation

**DOI:** 10.5811/cpcem.24973

**Published:** 2025-05-28

**Authors:** Brian Gooley, Kirk Hughes, Mark Gooley, Daniel Keyler, Richard Vetter, Jon Cole

**Affiliations:** * Minnesota Poison Control System, Minneapolis, Minnesota; † Mayo Clinic, Rochester, Minnesota; ‡ University of Minnesota, Minneapolis, Minnesota; § University of California Riverside, Riverside, California

**Keywords:** tarantula, Grammostola rosea, envenomation, muscle cramp

## Abstract

**Introduction:**

*Grammostola rosea* (Chilean rose tarantula) is a common exotic pet belonging to the Theraphosidae (tarantula) family. Case reports of theraphosid bites in adults commonly describe local tissue damage and local pain. Muscle spasms have also been described as a result of the bites but are rarer. We present a case of severe and persistent muscle spasms after a *G rosea* bite, which is uncommonly reported in the literature.

**Case Report:**

A 42-year-old woman was holding a *G rosea* tarantula when she was bit on the forearm. Within hours, severe local muscle cramping occurred. Due to worsening cramping, she initially presented to the emergency department the day after the bite, and again on the following day. She was admitted on her second visit and treated with diazepam, cephalexin, diphenhydramine, baclofen, cefpodoxime, doxycycline, prednisone, and topical hydrocortisone. Her laboratory testing was unremarkable, and while medical management may have mildly improved her symptoms, painful cramping persisted. After discharge, her paroxysmal muscle cramping continued for four weeks before completely resolving.

**Conclusion:**

While local tissue damage and pain are common, *G rosea* bites may lead to severe muscle cramping that persists for weeks. Standard laboratory testing may be completely normal in these cases. Muscle cramps may be persistent and are difficult to manage.

## INTRODUCTION

Theraphosidae (tarantulas) are popular exotic pets 1 and the most common culprit of pet arachnid envenomation. 2 Tarantulas, particularly the “New World” species of North, Central and South America, typically cause injuries to humans via their urticating hairs, which can penetrate into skin and mucous membranes leading to localized tissue reactions. 3 Bites from “New World” theraphosid spiders, although uncommon, can be fatal to small animals such as household pets and are generally described as benign in humans. 4 Nevertheless, rare cases of severe, delayed, prolonged, diffuse muscle cramps have been reported. 5

*Grammostola rosea* is a medium-sized tarantula native to Chile, Bolivia, and Argentina. Urticating hairs of *G rosea* appear rose-colored; hence, it is commonly known as the Chilean rose or rose hair tarantula. Known for their generally docile nature, *G rosea* are common among hobbyists and as a result are frequently sold around the world; 3 over 600,000 were traded in 2020 alone. 1 Like Theraphosidae in general, bites from *G rosea* are rare and poorly described in the medical literature. Here we report a case of *G rosea* envenomation resulting in severe, painful muscle cramping that lasted for several weeks.

## CASE REPORT

**Image 1:**
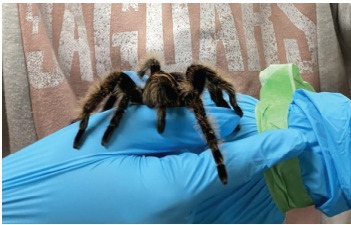
*Grammostola rosea*, the “Chilean rose” tarantula. (Both images shared by patient.)

A 42-year-old woman was bitten by a *G rosea* while at an exotic animal birthday party with her child. When her child was startled by the tarantula’s behavior, the patient took the tarantula from the child (Image 1) and was subsequently bitten on the forearm.

**Image 2:**
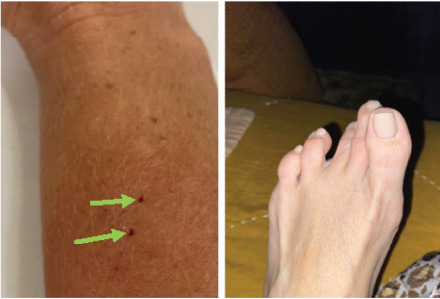
Bite puncture wounds denoted by green arrows (left) and pedal spasm (right).

The patient estimated the tarantula had its fangs inserted for 20–30 seconds and that it was making a “pulsing” motion with its body as she walked to the tarantula’s handler to ask for aid in removing it. The patient described the sensation as very painful with a feeling around the bite site as “scratchy, pinchy, and needles” around the two puncture wounds made by the bite (Image 2). Her arm then began to burn and ache, which persisted overnight.

The day after the bite her symptoms persisted. She called the exotic animal handler that morning and was recommended to treat the area with ice and ibuprofen, as would be done for a bee sting. Shortly after, while on a morning walk, the patient experienced severe, painful cramping in her feet that she rated as “10 of 10 pain” (Image 2). She had no history of muscle dystrophy or cramping. The patient is right-handed, and her past medical history was remarkable only for panic attacks, for which she was prescribed lorazepam as needed, but which she rarely used.

She returned home and drank an electrolyte solution with no improvement. Dorsiflexing her great toe seemed to help relieve the pain, while turning or twisting the leg exacerbated the cramps. Over the next few hours, she noticed that the cramps progressed proximally from her right ankle to her right thigh and hip flexors. The patient went to the local emergency department (ED), whereupon the regional poison center was consulted and recommended supportive care for muscle symptoms and local wound care. She was discharged after lab workup that included a normal basic metabolic panel and complete blood count. She was prescribed diazepam 2 milligrams (mg) every eight hours as needed and cephalexin 500 mg every six hours for seven days for muscle cramping and concern for local wound infection.

*CPC-EM Capsule*

What do we already know about this clinical entity?

*Effects beyond local tissue damage from tarantula bites are rare, and the management of these cases is mostly supportive*.

What makes this presentation of disease reportable?

*This is a case of prolonged muscle cramping that affected muscles far away from the site of envenomation. The cramping lasted for over a month*.

What is the major learning point?

*Tarantula envenomation may lead to debilitating muscle cramping that is difficult to manage with conventional medication for muscle cramps*.

How might this improve emergency medicine practice?

*Realizing that tarantula envenomation can lead to such prolonged symptoms will improve recognition of this rare condition and stimulate further study*.

Over the subsequent hours the patient developed worsening muscle spasms despite taking 0.5 mg of her lorazepam. The cramping progressed to her abdomen and prompted her return to the ED. She did not take the diazepam but did take the cephalexin as prescribed. The spasms were becoming more frequent with 1–3 minute paroxysms occurring in 15–20 minute intervals. During this ED visit, the erythema around the bite was noticed to be increasing in diameter, and she was admitted to internal medicine for pain control, intravenous fluids, and antibiotics.

On days 2–5 the patient was hospitalized with cramping occurring approximately every 30 minutes. Cramping frequency decreased on day 3 post-envenomation. She received diphenhydramine and baclofen during this hospital stay, which subjectively decreased the frequency of cramping. Throughout her stay she had been able to tolerate food and oral medications without difficulty. No further specialist consultations occurred during her hospital stay. She was discharged with baclofen, cefpodoxime, diphenhydramine, doxycycline, prednisone, and topical hydrocortisone, reporting that the severity had also decreased at this point.

On day 6 her cramping worsened again, accompanied by hand cramping that was exacerbated by use, similar to what she had experienced in her legs. The patient continued to take diazepam, diphenhydramine, and baclofen; however, unlike during hospitalization, these medications did not help her symptoms. Lying down also seemed to exacerbate spasms. On day 7 she started to notice myalgias.

Over the next two weeks the spasms continued but seemed to decrease in frequency. She noticed a burning pain in her legs in multiple locations and described other sensations such as neuropathic pain in her feet that made it painful to walk. She noticed muscle cramping in her face two weeks after the bite, which recurred on two other occasions. By week four her symptoms had completely improved, and no further cramping occurred.

## DISCUSSION

While local wound reactions are the most common result in human tarantula exposures, systemic and regional symptoms do occur with tarantula envenomation. 6 These symptoms can occur after envenomation from a wide variety of tarantula species. Symptoms may be delayed by hours and last for several weeks, similar to the case we present here. 7 Burning, pain, swelling, and localized muscle cramping are common after these bites. One study suggests that 23% of patients may develop muscle cramps and 12.7% of bites can lead to pain affecting several parts of the body. 2 Because there is a paucity of confirmed cases in the medical literature, guidance from medical professionals is heavily dependent upon sources such as internet forums and other non-traditional resources in addition to the few published cases.

Our patient had no obvious serum electrolyte derangements that would have contributed to dysfunctional nerve conduction or muscle contraction. There was no evidence of rhabdomyolysis, which has also been absent in other case reports. 8 In other case reports of envenomation by *Lampropelma nigerrimum (*Sangihe Island tarantula*)*, severe, spreading muscle spasms and trismus occurred. In contrast to our case, elevations in creatine kinase were seen; however, as in our case electrolytes were normal. 8 In those cases, one patient had symptoms for seven days and the others were lost to follow-up. Although literature is limited, it appears muscle spasms can occur for days to weeks after tarantula envenomation.[Table t1-cpcem-9-285]

The mechanism of muscle-spasm toxicity from theraphosid envenomation is not well understood, but it is theorized that a direct toxic effect from the venom is the etiology. Tarantula venom in general is composed of organic peptides and molecules, none of which have been studied to completely explain the effects of muscle cramping in humans. The GTx1–15 toxin is an inhibitor cystine knot peptide found in *G rosea* venom and has been shown to inhibit both low-voltage activated Ca_v_3.1 calcium channels and sodium channels of Na_v_1.3 and Na_v_1.7 subtypes. 9 Furthermore, the degradation of this toxin is prolonged, showing little degradation at 24 hours in the same study. Both sodium- and calcium-channel dysfunction could affect muscle contractions and potentially lead to spontaneous or prolonged depolarization. Difficulties identifying the exact mechanism and cause of severe muscle cramping is likely related to varying amounts of venom quantity and composition based on individual spider species, diet, and geographic distribution.

In this case, our patient felt very little relief from the various medications administered for treatment of muscle spasms. Patients in other published cases have been treated with benzodiazepines, magnesium, and calcium; however, the effectiveness of these treatments has been variable and unproven. 8 In a mouse study assessing venom-induced toxicity from tarantulas of the *Poecilotheria* genus, mice were injected with sub-lethal doses of venom and were split into groups that received atropine, chlorpromazine, chloropyramine, diazepam, ethanol, flupirtine, haloperidol, ketotifen, lamotrigine, oxcarbazepine, tolperisone, xylazine, and calcium chloride via various administration routes; chlorpromazine was the only medication found to reduce muscle cramping following administration of venom. 10 Further research to delineate the most effective treatments for Theraphosidae envenomation is needed.

## CONCLUSION

Envenomation from *Grammostola rosea* may lead to significant and prolonged muscle cramping. The mechanism of toxicity is poorly understood, and no specific therapy has been established as effective. Further research is needed into medical treatments to effectively manage these venom-induced muscle spasms. Patients should be counseled that delayed muscle cramps may occur in a delayed fashion and can potentially last for weeks.

## Figures and Tables

**Table t1-cpcem-9-285:** Chart of laboratory values during patient ED visits and hospital admission.

Day of and following the bite	Day 1	Day 2	Day 3	Day 4	Reference Range
Na (mmol/L)	134	137			134–144
K (mmol/L)	4.2	4.3			3.5–4.2
Cl (mmol/L)	102	108			96–106
HCO_3_ (mmol/L)	24	22			23–29
Glucose (mg/dL)	135	92			65–99
Anion Gap (mmol/L)	8	7			4–12
Calcium (mg/dL)	9.2	8.5			8.7–0.2
BUN (mg/dL)	16	14			6–24
Creatinine (mg/dL)	0.92	0.71	0.80	0.72	0.76–1.27
ALT (U/L)	17				0–44
AST (U/L)	25				0–40
Magnesium (mg/dL)	2.0				1.7–2.2
Phosphorus (mg/dL)	2.8				2.5–4.6
CK, Total (U/L)	124	90			30–170
ESR (mm/hr)			16		<15
CRP (mg/dL)			0.7	0.5	<1
WBC (K/mcL)	8.8	6.5	6.0		4.5–11
Hemoglobin (g/dL)	13.6	12.5	12.7		12–15
Platelets (K/mcL)	358	311	322		150k–450k

Abbreviations: *Na*, sodium; *K*, potassium; *Cl*, chloride; *HCO_3_*, bicarbonate; *BUN*, blood urea nitrogen; ALT, alanine aminotransferase; *AST*, aspartate aminotransferase; *CK*, total creatine kinase; *ESR*, erythrocyte sedimentation rate; *CRP*, C-reactive protein; *WBC*, white blood cell count.
